# Genetic Control of Fruit-to-Bean Ratio and Mass-Based Metrics for Processing Efficiency in *Coffea canephora*

**DOI:** 10.3390/plants15091407

**Published:** 2026-05-05

**Authors:** Jéssica Almeida Jorge, Cleidson Alves da Silva, Deurimar Herênio Gonçalves Júnior, Ivoney Gontijo, Rodrigo Barros Rocha, Eveline Teixeira Caixeta, Weverton Pereira Rodrigues, Fábio Luiz Partelli

**Affiliations:** 1 Northern Espírito Santo University Center (CEUNES), Federal University of Espírito Santo (UFES), São Mateus 29932-900, ES, Brazil; 2 Experimental Field of Patrocínio, Agricultural Research Company of Minas Gerais (EPAMIG), Patrocínio 38740-000, MG, Brazil; 3 Agricultural Research Corporation (Embrapa Coffee), Brasília 70770-901, DF, Brazil; 4 Capixaba Institute for Research, Technical Assistance and Rural Extension (INCAPER), Vitória 29052-010, ES, Brazil; 5 Institute of Biotechnology Applied to Agriculture (Bioagro), Federal University of Viçosa (UFV), Viçosa 36570-900, MG, Brazil; 6 Center for Agricultural Sciences (CCA), State University of the Tocantins Region of Maranhão (UEMASUL), Imperatriz 65900-001, MA, Brazil

**Keywords:** *Coffea canephora*, heritability, fruit yield efficiency, genetic diversity, multivariate analysis, bean proportion

## Abstract

The expanding use of Conilon and Conilon × Robusta hybrids in Brazilian coffee cultivation contrasts with the scarcity of information on the genetic variability underlying their physical yield attributes. This study quantified genetic variability and estimated genetic parameters for fresh fruit mass and volume, fruit-to-bean ratio, and bean and husk proportions in 48 *Coffea canephora* genotypes, compared the discriminatory power of gravimetric and volumetric metrics in classifying processing efficiency, and identified genotypes combining high bean proportion and genetic divergence for use in breeding programs. A randomized complete block design with three replications was used. The fruit mass required to produce a 60 kg bag of processed coffee (FMM/bag) ranged from 205.29 to 251.46 kg bag^−1^, representing an 18% difference between the most and least efficient groups identified by the Scott-Knott test. High heritability was found for bean proportion (90.74%) and FMM/bag (84.58%), confirming strong genetic control over fruit-to-bean yield. Mass-based metrics showed greater discriminatory power than volumetric ones, forming four distinct groups versus two. Conilon genotypes tended toward greater yield efficiency. The observed variation indicates exploitable genetic variability for selective gains, with direct implications for crossing strategies and post-harvest processing optimization in *C. canephora*.

## 1. Introduction

Processing efficiency is a key determinant of profitability in coffee production, as bean yield directly influences costs, industrial capacity, and the final product value [1]. In *Coffea canephora*, bean yield as a function of harvested fruit quantity is highly variable, with an average conversion close to 27% of ripe fruit weight per processed bag, although the described genetic potential ranges from 51% to 62% of seed mass per fruit [2].

Traditionally, bean yield estimation in *C. canephora* cultivation is performed by quantifying the volume of ripe fruit required to produce a 60 kg bag of processed coffee. However, this volume-based measurement has limitations, since fruit size and density can vary widely among genotypes. In contrast, the use of ripe fruit mass per bag of processed coffee has proven more precise. For example, Partelli et al. [2] demonstrated that the mass-based metric has superior discriminatory power, revealing genetic variation that volume-only measurements would be unable to detect.

Fruit-to-bag conversion efficiency results from the interaction between physiological processes and structural fruit attributes. Mass reflects source-sink dynamics and endosperm reserve accumulation [3], while volume is associated with pericarp development and mesocarp cell expansion [4]. The dissociation between mass and volume suggests variation in fruit structural density, with direct implications for the proportion of economically usable tissue.

Beyond differences in mass and volume, the dry matter proportion of the fruit provides a complementary dimension for interpreting conversion efficiency and the structural quality of the harvested material. Indeed, dry matter content serves as an indicator of endosperm maturation and consolidation, helping to control variation associated with harvest stage [5,6]. Its direct relationship with physical yield, however, remains poorly explored in broad germplasm populations.

Genetic variability in fruit structural attributes of *C. canephora* remains poorly quantified under controlled experimental designs. Most studies conducted to date have focused on agronomic traits or beverage quality. This bias in breeding programs may have practical consequences: genotypes superior in conversion efficiency may be discarded in selections based exclusively on productivity, since a highly productive genotype with low bean proportion may yield less processed product than a moderate genotype with high conversion efficiency. In Brazil, where hybrids between Robusta and Conilon have been gaining ground in coffee cultivation, the study of physical yield attributes becomes particularly relevant, especially when evaluated under direct competition in the main *C. canephora* producing region of the country.

In this context, this study aimed to quantify genetic variability and estimate genetic parameters for fresh fruit mass and volume, fruit-to-bean ratio, and bean and husk proportions in 48 *C. canephora* genotypes, as well as to compare the discriminatory power of gravimetric and volumetric metrics in classifying processing efficiency, characterize the structure of genetic divergence through multivariate analysis, and identify genotypes combining high bean proportion and genetic divergence for strategic use in breeding programs oriented toward yield efficiency in drying and processing. The hypothesis of this study is that grain yield efficiency in *C. canephora* is primarily determined by variation in fruit structural composition, particularly by bean proportion, which explains the superior discriminatory capacity of mass-based metrics relative to volumetric ones.

## 2. Results

### 2.1. Descriptive Statistics and Analysis of Variance

The results showed that genetic variability exists among *C. canephora* genotypes for processed coffee yield. Analysis of variance revealed a significant genotype effect (*p* < 0.05) for all variables related to physical yield, except for the fruit volume-to-mass ratio (FVM FMM^−1^), which showed no difference among genotypes. Experimental coefficients of variation ranged from 2.45% to 7.82% (Table 1), indicating stability of estimates among plots.

The overall mean for fresh fruit mass per bag of processed coffee (FMM bag^−1^) was 223.84 kg bag^−1^. The volume required per bag (FVM bag^−1^) had a mean of 356.25 L bag^−1^, while the fruit mass-to-processed bean mass ratio (FMM PBM^−1^) was 3.73. The mean bean proportion was 55.33%, ranging from 44.55% to 61.61% among genotypes, and the mean husk proportion was 44.67% (Table 1).

The genotypic variance (σ^2^g) was expressed for FMM bag^−1^ (189.95), FVM bag^−1^ (414.39), FMM PBM^−1^ (0.053), % Bean (5.99), and % Husk (5.99), while environmental variance (σ^2^e) was relatively low for these same variables, reflecting high experimental control (Table 2). In contrast, for FVM FMM^−1^, genotypic variance was null (σ^2^g = 0), and the observed variation was due exclusively to environmental factors (σ^2^e = 0.0052).

Heritability on a family mean basis (h^2^) ranged from 62.93% for FVM bag^−1^ to 90.74% for bean and husk proportions, confirming that most of the observed phenotypic differences are attributable to genotypic differences, except for FVM FMM^−1^. The CVg/CVe ratio confirms selection potential, with values above 1.0 for FMM bag^−1^ and FMM PBM^−1^, while variables such as FVM bag^−1^ showed CVg/CVe below 1.0, reflecting a smaller genotype effect relative to environmental variation (Table 2).

### 2.2. Fruit-to-Bean Conversion Efficiency

**Figure 1:**
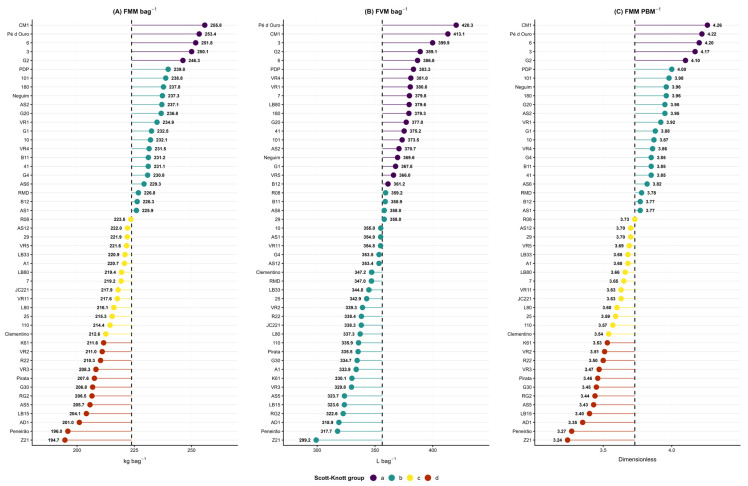
Performance of *Coffea canephora* genotypes for: (**A**) fruit mass per processed bag (FMM bag^−1^, kg bag^−1^), (**B**) fruit volume per processed bag (FVM bag^−1^, L bag^−1^), and (**C**) fruit mass-to-processed bean mass ratio (FMM PBM^−1^, dimensionless). Points represent genotype means, colors indicate groups formed by the Scott-Knott test (5%), and the dashed circular line corresponds to the overall mean of each variable.

Genotype classification based on FMM bag^−1^ resulted in the formation of four distinct groups by the Scott-Knott test (Figure 1). The Scott-Knott test was preferred over pairwise comparison methods such as Tukey and Duncan because it partitions treatment means into non-overlapping homogeneous groups without inflation of type I error, a property particularly relevant in experiments with a large number of treatments. With 48 genotypes, pairwise methods would generate excessive fragmentation and overlapping groups, compromising the practical interpretation of results [7]. The group with the highest mass requirement had a mean of 251.46 kg bag^−1^, comprising five genotypes: four from the Robusta group (G2, 03, 06, and Pé de Ouro) and one from the Conilon group (CM1). The most efficient group had a mean of 205.29 kg bag^−1^, corresponding to an approximately 18% reduction in the fresh fruit mass required to obtain one processed bag. This group comprised twelve genotypes, of which five belong to the Conilon variety (Z21, Peneirão, AD1, Pirata, and K61) and seven are Robusta hybrids (LB15, AS5, RG2, G30, VR3, R22, and VR2).

### 2.3. Bean Proportion and Its Association with Yield Metrics

**Figure 2:**
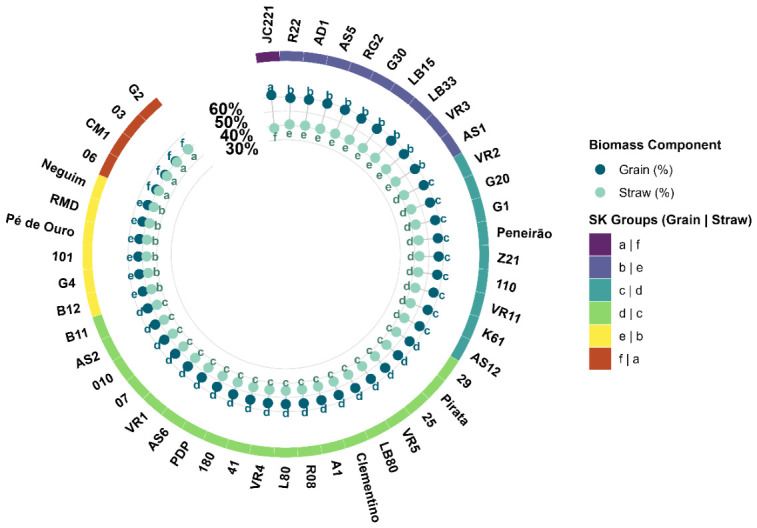
Percentage distribution of bean and husk in 48 *Coffea canephora* genotypes. The outer ring and colored letters represent the statistical grouping by the Scott–Knott test (*p* < 0.05). Means followed by the same letter, within each component (bean or husk), do not differ from each other.

Bean and husk proportions in the fruits formed six distinct groups (Figure 2). The hybrid genotype JC221 was allocated alone in the superior group, with 61.61% bean proportion. The second group with the highest bean percentage means was also composed of hybrid coffee plants, with a mean of 58.62% bean and 41.38% husk. All Conilon genotypes were allocated to intermediate groups for bean and husk percentage in the fruit, with the exception of genotype CM1, which together with three other hybrid genotypes (G30, G20, and 101) formed the group with the lowest bean percentage means and highest husk percentage in the fruit. The range between extremes represented a relative variation of approximately 38%.

**Figure 3:**
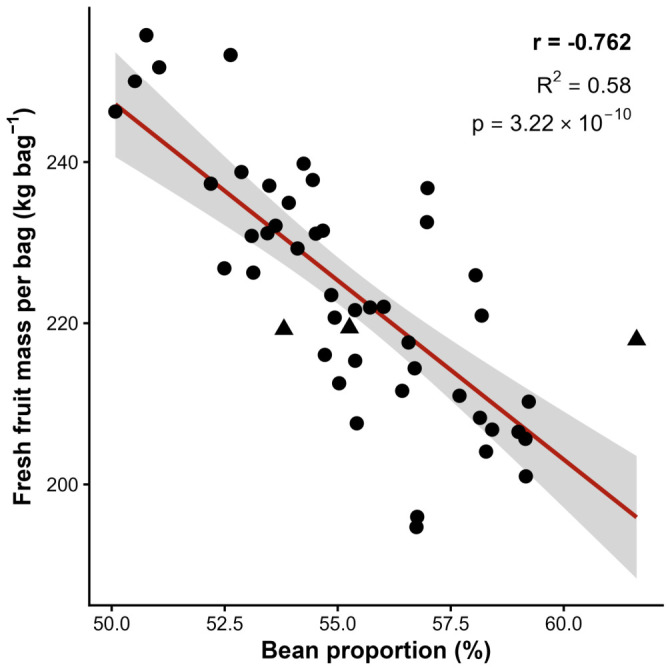
Linear regression between bean proportion (%) and fresh fruit mass per processed bag (FMM bag^−1^, kg bag^−1^) across 48 *Coffea canephora* genotypes. Each point represents a genotype mean estimated by the marginal means method (EMMs). The shaded area indicates the 95% confidence interval of the regression line (r = −0.762, R^2^ = 0.580, *p* < 0.001).

The relationship between bean proportion and FMM bag^−1^ was inverse and statistically significant (r = −0.762, *p* < 0.001), with linear regression explaining 58% of the observed variation in fruit mass requirements (R^2^ = 0.580; Figure 3). The regression coefficient indicated that each additional percentage point in bean proportion reduced fresh fruit mass per bag by approximately 4.4 kg bag^−1^, a magnitude with direct practical implications for harvest logistics and post-harvest processing capacity.

### 2.4. Clustering Structure and Phenotypic Divergence

**Figure 4:**
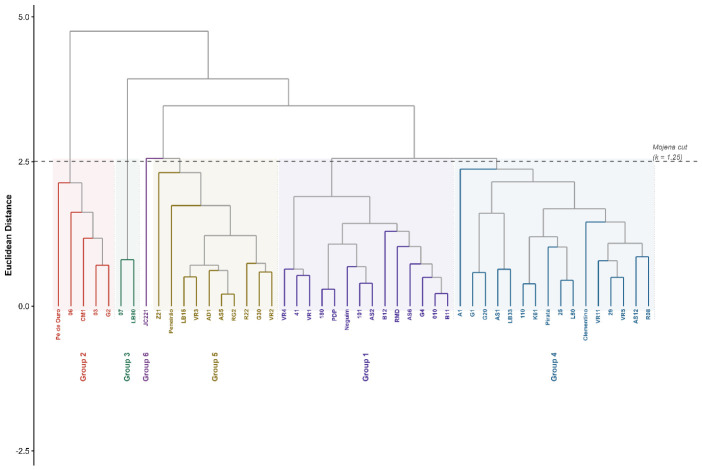
Dendrogram of genetic dissimilarity among 48 *Coffea canephora* genotypes obtained by UPGMA clustering analysis with Euclidean distance. The grey dashed line indicates the cutoff point adopted for group definition. Grey lines represent the hierarchical connections of the dendrogram above the Mojena cutoff point.

Hierarchical clustering by the UPGMA method, considering a cutoff point of 52.80% dissimilarity (k = 1.25), resulted in the formation of six distinct groups. The cophenetic correlation coefficient was 0.74, indicating a moderate fit between the original dissimilarity matrix and the dendrogram (Figure 4). Clustering resulted in six phenotypically distinct groups. Three groups were composed exclusively of hybrids (Groups 1, 3, and 6), while Groups 2, 4, and 5 comprised both Conilon and hybrid genotypes simultaneously.

Group 6 was composed of genotype JC221, isolated from the others by the highest bean percentage recorded in the study (61.61%, group “a” by the Scott-Knott test). Group 5 concentrated ten genotypes with the highest bean proportions after JC221 (groups “b” and “c”), comprising three Conilon (AD1, Peneirão, and Z21) and seven hybrids (AS5, G30, LB15, R22, RG2, VR2, and VR3). Group 4, the most numerous with 17 genotypes, showed bean proportions distributed among groups “b” and “d” of the Scott-Knott test, comprising four Conilon (Clementino, K61, L80, and Pirata) and 13 hybrids. Groups 1 and 3, composed entirely of hybrids, showed bean proportions in groups “c” to “e”. Group 2 comprised the genotypes with the lowest bean proportion (groups “e” and “f”), being formed by four hybrids (03, 06, G2, and Pé de Ouro) and one Conilon (CM1). Conilon genotypes were present in Groups 2, 4, and 5, ranging from the worst to the best performing group for bean proportion. The same pattern was observed for hybrids, present in all six groups.

**Figure 5:**
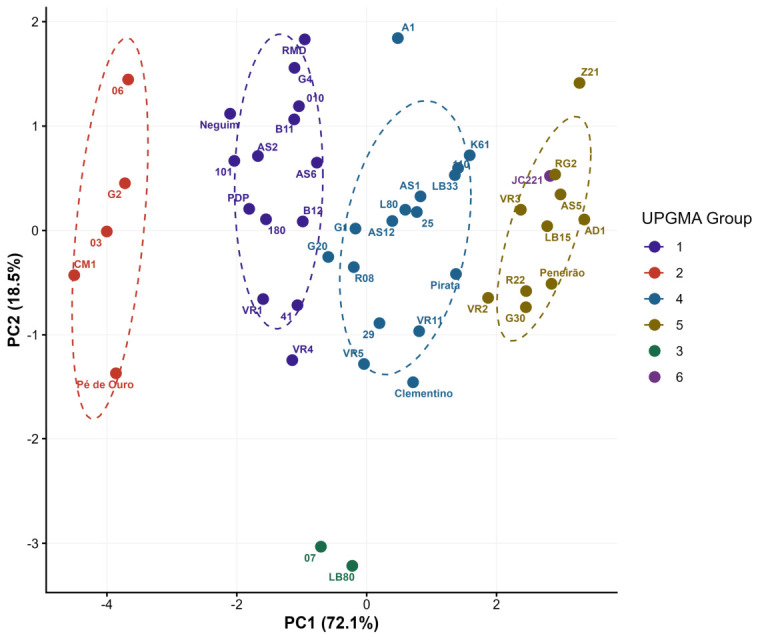
Principal component analysis (PCA) of 48 *Coffea canephora* genotypes based on standardized phenotypic means for fruit mass per bag (FMM bag^−1^), fruit volume per bag (FVM bag^−1^), fruit mass-to-bean mass ratio (FMM PBM^−1^), and bean and husk proportions. Colors indicate UPGMA clustering groups. Dashed ellipses represent the 68% confidence region for groups with *n* ≥ 3 genotypes. PC1 and PC2 together explained 90.6% of the total variance.

Principal component analysis corroborated the grouping structure identified by UPGMA clustering. The first two principal components explained 90.6% of total phenotypic variance (PC1 = 72.1%; PC2 = 18.5%), with PC1 driven primarily by mass-based metrics and bean proportion (Figure 5). Genotypes allocated to Group 2 by UPGMA—those with the lowest bean proportion—were consistently projected at the negative extreme of PC1, while genotypes from Group 5, with the highest bean proportions after JC221, occupied the positive extreme. Groups 3 (07 and LB80) and 6 (JC221) remained isolated in the PCA space, confirming their phenotypic distinctiveness relative to the remaining germplasm.

## 3. Discussion

### 3.1. Fruit-to-Bean Conversion Efficiency and the Physical Nature of the Descriptors

The fresh fruit mass required to obtain one processed bag varied by 22% between the extreme groups formed by the Scott-Knott test, from 205.29 to 251.46 kg bag^−1^. This range, recorded under experimental coefficients of variation between 2.45% and 7.82%, reflects genotypic differences in the proportion of reproductive and storage tissues, not harvest variation. Variability of comparable magnitude for physical yield attributes in *C. canephora* was documented by Partelli et al. [2], who evaluated 43 genotypes and identified a similar range for the fruit-to-bean ratio, and by Dalazen et al. [7] and Lopes Júnior et al. [5], in Conilon and Amazonian Robusta populations, respectively [5,8,9,10].

The mass-based metric separated the 48 genotypes into four groups, while volume differentiated only two. The CVg/CVe ratio above 1.0 for FMM bag^−1^ and FMM PBM^−1^ confirms that variation in these metrics is predominantly genotypic. For FVM FMM^−1^, genotypic variance was null (σ^2^g = 0), and all observed variation was attributable to environmental factors, which is consistent with the geometric nature of volume, whose estimation by water displacement is sensitive to variations in turgor and sample compaction [5,6,11]. The absence of genotypic effect for FVM FMM^−1^ indicates that mass and volume are proportional among genotypes, meaning that the mean fruit density is uniform across the evaluated set. Efficiency differences among genotypes therefore arise from the balance between endosperm and pericarp, consolidated during fruit development [12], and not from variations in residual water content [13].

It should be noted that the absence of genotypic variation for FVM FMM^−1^ may reflect genuine biological uniformity in fruit structural density across the evaluated germplasm, or alternatively, insufficient statistical power to detect small genetic effects given the sample size and number of replications. The geometric nature of volumetric measurements, sensitive to compaction and turgor variation during water displacement assays, may also have contributed to inflated environmental variance relative to genotypic signals.

The lower heritability of FVM bag^−1^ (h^2^ = 62.93%) does not eliminate its operational relevance. A difference of more than 40 L bag^−1^ between extreme groups has a direct impact on the sizing of harvest containers, drying capacity, and post-harvest logistics in manual systems [14]. The distinction between the two scales has a practical implication: FMM bag^−1^ is the most robust descriptor for identifying superior genotypes in breeding programs, while FVM bag^−1^ retains utility for physical planning of the production chain. The indiscriminate use of only one of the metrics compromises both selection and logistical decisions.

### 3.2. Genetic Control of Bean Proportion and Response to Selection

Variance components were obtained by the method of moments, which assumes genotypes as a random sample of a broader population for the purpose of component estimation. The resulting heritability estimates therefore have a mixed inferential character: they are technically valid as predictors of selective response within the evaluated germplasm, but their extrapolation to the species requires caution, given that the evaluated set is not a random sample of *C. canephora* diversity [14,15]. Heritability on a family mean basis was high for most evaluated variables: 84.6% for FMM bag^−1^ and FMM PBM^−1^, and 90.7% for bean and husk proportions. These values indicate that most of the observed phenotypic variation has a genotypic origin, which is a favorable condition for selection. The CVg/CVe ratio above 1.0 for FMM bag^−1^, FMM PBM^−1^, and bean proportion reinforces the selective potential of these variables [9,14,15].

Bean proportion simultaneously showed the highest heritability in the set (90.7%), the lowest experimental coefficient of variation (2.45%), and a CVg/CVe of 1.81, positioning it as the most efficient descriptor for genotypic discrimination among those evaluated. The elevated heritability of bean proportion likely reflects the genetic control over endosperm development during a phenologically constrained window of fruit filling. Unlike fruit volume, which depends on mesocarp cell expansion and is sensitive to water availability and temperature fluctuations during ripening, the dry mass fraction allocated to the endosperm is consolidated earlier in fruit ontogeny and under tighter metabolic regulation [12]. Under uniform irrigation management as applied in this experiment, environmental sources of variation for bean proportion are further minimized, which explains the low residual variance (σ^2^e = 0.61) relative to the genotypic component (σ^2^g = 5.99). This biological basis reinforces bean proportion as a selection criterion with predictable response across cycles within similar management conditions [16]. Gaspari-Pezzopane et al. [17], evaluating *Coffea* germplasm, recorded intrinsic yield ranging from 30% to 64%, an interval within which the genotypes of the present study were distributed, from 44.55% to 61.61%. Genotype JC221 reached the highest value (61.61%), close to the upper limit documented for the genus.

The absence of genotypic variation for FVM FMM^−1^ (σ^2^g = 0, h^2^ undetermined) indicates that this variable does not respond to selection under the evaluated conditions and should not be incorporated into selection indices for yield efficiency. In contrast, the combination of high heritability and elevated CVg/CVe for bean proportion suggests that consistent selective gains are feasible from the evaluated germplasm, without the need for contrasting environments for trait expression [8,18].

### 3.3. Diversity Structure and Strategic Implications

The cophenetic correlation coefficient of 0.74 indicates a moderate fit between the original dissimilarity matrix and the dendrogram, exceeding the 0.70 threshold frequently considered acceptable for hierarchical clustering [19], based on the cophenetic correlation framework originally proposed by Sokal and Rohlf [20].

Three groups were composed exclusively of hybrids (Groups 1, 3, and 6), while Groups 2, 4, and 5 comprised Conilon and hybrid genotypes simultaneously. No group was composed exclusively of Conilon genotypes. Group 5, which concentrated the genotypes with the highest bean proportion after JC221, included three Conilon (AD1, Peneirão, and Z21) and seven hybrids. Group 2, the worst performing group, comprised four hybrids and only one Conilon (CM1). This distribution indicates that the variety group does not delimit performance for yield efficiency in the evaluated set.

Analysis of genotypes common to the present study and the works of Silva et al. [9] and Silva et al. [21] showed that hybrids with greater Robusta introgression (LB33, LB80, AS6, R22, AS2, LB15) were allocated to distinct groups in the dendrogram, with no consistent clustering pattern by molecular origin. The same was observed for genotypes with greater Conilon introgression (AS1, AS5, RMD, AS12). Oliveira et al. [22] demonstrated that the distinction between Conilon and Robusta requires anchoring in discriminant molecular markers, and that a minimum set of ten markers already allows differentiation with high accuracy. The absence of correspondence between molecular identity and phenotypic clustering for yield reinforces that morphoagronomic descriptors alone underestimate the population architecture of *C. canephora*.

The isolated position of JC221 in Group 6, determined by the highest bean proportion in the set (61.61%), does not imply overall superiority. Attributes such as tolerance to biotic and abiotic stresses and beverage quality were not evaluated and must be considered before any recommendation for use as a parental genotype. The dissociation between univariate and multivariate clustering, in turn, confirms that selection based on a single descriptor captures only a fraction of the available variability [23,24].

Some limitations of the present study warrant explicit acknowledgment. The experiment was conducted in a single commercial environment over two crop years, which precludes formal quantification of genotype × environment interaction for the evaluated yield metrics, a recognized limitation in perennial crop breeding, where multi-environment trials are required to distinguish broad from specific adaptation [24,25]. The genotypic variability detected for bean proportion and gravimetric metrics is nonetheless consistent across both harvests and indicates potential for selective response within this environment. Additionally, the evaluated set of 48 genotypes, while representative of the main genetic groups cultivated in the principal *C. canephora* producing region of Brazil, is not a random sample of the species’ diversity, which restricts extrapolation of the estimated genetic parameters to the broader population. Finally, traits such as tolerance to biotic and abiotic stresses and beverage quality were not evaluated and must be considered in integrated selection indices before any genotype is recommended for use as a parent in crossing programs. The incorporation of multi-environment trials, molecular data, and quality metrics will allow identification of informative genetic contrasts and translation of the observed variability into measurable selective gains.

## 4. Materials and Methods

### 4.1. Experimental Area and Edaphoclimatic Conditions

The experiment was conducted in a commercial cultivation area located in the municipality of Jaguaré, northern Espírito Santo state, Brazil (18°59′33″ S, 39°55′07″ W; approximate altitude of 50 m). The regional climate is classified as Aw according to Köppen, characterized by a rainy season in summer and a well-defined dry period in winter, with a mean annual temperature close to 23.5 °C and low frost occurrence [26,27]. The soil of the experimental area is classified as a typical dystrophic Yellow Latosol, according to the Brazilian Soil Classification System [28], correlated to a Ferralsol (Dystric, Xanthic) according to the World Reference Base for Soil Resources [29].

### 4.2. Meteorological Data

The meteorological data were obtained from an automatic weather station located in the municipality of Jaguaré [30], covering the period from August 2022 to July 2024. Mean air temperature, monthly accumulated rainfall, relative air humidity, reference evapotranspiration, and daily thermal amplitude were monitored. Mean monthly temperature varied throughout the period, with values exceeding 26 °C in the warmest months, while rainfall was concentrated between November and February, peaking at approximately 480 mm in December 2022, and a dry period between April and October. Daily thermal amplitude ranged from 6 °C to 11 °C, with maximum values recorded in February (10 °C) and March 2024 (11 °C). These data supported the climatic characterization of the experimental period and the management of supplemental irrigation adopted in the experiment.

### 4.3. Plant Material and Experimental Design

A total of 48 *C. canephora* genotypes were evaluated, comprising eight plants from the Conilon variety group and 40 hybrids between Conilon and Robusta (Table 3). Among the hybrids, 39 originated from the state of Rondônia, and genotype A1 from Espírito Santo, which was recently identified as a hybrid [22]. The Conilon genotypes originated from Espírito Santo and Bahia [31,32].

Planting was carried out in April 2021, at a spacing of 3.0 m between rows and 0.6 m between plants, resulting in a density of 5556 plants ha^−1^. Plants were trained with two orthotropic stems, following the practice adopted in commercial *C. canephora* systems. The experimental design was a randomized complete block design, with 48 treatments and three replications. Each plot consisted of seven plants, with the five central plants considered useful, in order to minimize border effects.

### 4.4. Irrigation Management and Cultural Practices

The experiment was conducted under drip irrigation, with water depths adjusted to evapotranspiration and phenological stage in order to avoid water stress during flowering, fruit filling, and ripening. Fertilization, phytosanitary management, and weed control followed regional technical recommendations for *C. canephora* in Espírito Santo, ensuring agronomic conditions compatible with commercial systems and minimizing management variation among plots.

### 4.5. Fruit Processing and Physical Traits Evaluation

Harvesting was carried out in June 2023 and June 2024, covering two consecutive crop years. Data from both harvests were averaged per genotype prior to statistical analysis, reducing within-genotype temporal variation and improving the reliability of trait estimates. Harvest was performed manually by selective stripping, ensuring uniformity of ripening stage. For each genotype, three independent samples of 25 ripe fruits per plot were collected, totaling 75 fruits per genotype in each replication. Samples were obtained from branches distributed across different strata of the plant and immediately taken to the laboratory for processing. This sampling protocol follows the methodology validated by Partelli et al. [2] for *C. canephora* physical yield traits, in which samples of 25 fruits collected from multiple plant strata have been shown to adequately represent within-plot variability. The collection across three plant strata per plot, combined with three replications per genotype, resulted in 75 fruits evaluated per genotype per harvest, with experimental coefficients of variation between 2.45% and 7.82% confirming adequate precision of the estimates.

Fruits were individually weighed on a precision analytical balance (0.001 g) to determine fresh weight. The volume occupied by the ripe fruits was determined by the volumetric displacement method, using a 100 mL graduated cylinder filled with distilled water. The difference between the initial volume and the volume after fruit immersion corresponded to the total volume of the sample. Subsequently, samples were dried in a forced-air oven at 50 °C until constant mass, a criterion reached when the mass variation between two consecutive weighings, performed at 24 h intervals, was less than 0.5%.

After complete drying, manual husking was performed to separate the bean (dry endosperm) and husk (pericarp, mesocarp, and endocarp), with each fraction weighed independently on an analytical balance. The fruit-to-bean ratio was expressed as the ratio between the dry mass of the whole fruit and the dry mass of the processed bean. From these data, the variables fresh fruit mass per 60 kg bag of processed coffee (FMM bag^−1^) and fresh fruit volume per 60 kg bag of processed coffee (FVM bag^−1^) were calculated. For FMM bag^−1^, fresh fruit mass and processed bean mass (PBM) were considered. For FVM bag^−1^, fruit volume and PBM were considered.

Bean and husk proportions were calculated based on the dry mass of the fruit, as the percentage fraction of each component relative to the total dry mass of the dried fruit:

$$Bean\;(\%)=(\frac{bean\;mass}{dry\;fruit\;mass})\times 100$$

$$Husk\;(\%)=(\frac{husk\;mass}{dry\;fruit\;mass})\times 100$$

Additionally, overall physical yield was estimated by a conversion index integrating the relationship between fresh fruit mass from the field, dried fruit mass, processed bean fraction, and correction to 12% moisture content (outturn index):

$$Outturn\;index=\left(\frac{m_{dry\;cherry}}{m_{from\;field}}\right)\times \left(\frac{m_{beans}}{m_{beans}+m_{hull}}\right)\times F_{moist12\%}\times 100$$

where the outturn index was estimated by the relation between the weight of the dry cherry coffee ($m_{dry\;cherry}$) and the weight of the coffee from the field ($m_{from\;field}$), together with the relation between the weight of the coffee beans (m beans) and the weight of the dried fruits ($m_{beans}+m_{hull}$), corrected to 12% moisture ($F_{moist12\%}$).

### 4.6. Statistical Analysis

The data were initially subjected to exploratory analysis to verify outliers, residual normality (Shapiro–Wilk test), and variance homogeneity (Bartlett test). Once parametric assumptions were met, analysis of variance (ANOVA) was performed according to the statistical model:

$$Y_{ij}=\mu +G_{i}+B_{j}+\varepsilon_{ij}$$

where $Y_{ij}$ represents the observed value of the i-th genotype in the j-th block; μ is the overall mean; $G_{i}$ corresponds to the random effect of genotype (i = 1, …, 48) with Gi~N(0, σ^2^g); $B_{j}$ to the fixed effect of block (j = 1, 2, 3); and $\varepsilon_{ij}$ to the experimental error, assumed as $\varepsilon_{ij}\sim N(0,\;\sigma^{2})$.

Variance components were estimated by the method of moments from the ANOVA expected mean squares, treating genotypes as random effects. Heritability on a family mean basis (h^2^) was estimated as:

$$h^{2}=\frac{\sigma_{g}^{2}}{\sigma_{g}^{2}+\sigma_{e}^{2}/r}$$

where $\sigma_{g}^{2}$ is the genotypic variance, $\sigma_{e}^{2}$ the residual variance, and r the number of replications. In addition, the genotypic coefficient of variation (CVg) and the CVg/CVe ratio were calculated to assess relative genetic variability and the selection potential of the descriptor.

Significance of effects was tested by the F-test at 5% probability. When significant differences were detected, means were grouped by the Scott–Knott test at 5% probability. This procedure was adopted due to the large number of treatments (48 genotypes), aiming to maximize discrimination among homogeneous groups and reduce the excessive fragmentation typical of pairwise comparison methods.

For phenotypic diversity analysis, adjusted means (EMMs) were first obtained for each genotype, considering the linear model described above. EMMs were estimated using the estimated marginal means method (emmeans), which allows genotypes to be compared by removing the systematic influence of blocks and ensuring adjusted and comparable estimates among genotypes.

The adjusted means of the multivariate variables were then standardized by z-score, in order to eliminate scale differences and ensure that all variables contributed equally to the dissimilarity measure. The phenotypic dissimilarity matrix was calculated using the mean Euclidean distance, given by:

$$d_{ij}=\sqrt{\frac{1}{p}\sum_{k=1}^{p}(x_{ik}-x_{jk}{)}^{2}}$$

where $d_{ij}$ represents the dissimilarity between genotypes i and j, p the number of evaluated variables, and $x_{ik}$ the standardized value of variable k in genotype i.

Hierarchical clustering was performed using the UPGMA method (Unweighted Pair Group Method with Arithmetic Mean). The dendrogram cutoff point was determined by the Mojena [33] method:

$$d_{c}=\overset{ˉ}{d}+k\cdot s_{d}$$

where $d_{c}$ is the critical fusion distance, $\overset{ˉ}{d}$ the mean of clustering distances, s d the standard deviation of these distances, and k = 1.25, the value adopted in this study. All analyses were implemented in the RStudio integrated development environment (IDE), using the R programming language, version 4.5.1 [34]. The ggplot2 package [35] was used for graph generation.

## 5. Conclusions

Significant genetic variability exists for yield traits in *C. canephora*. High heritability estimates confirmed expressive genetic control, indicating prospects for selective gains in processed bean yield. Multivariate analysis reinforced dissimilarity among genotypes, forming distinct groups that can be used in plant breeding programs.

Mass-based metrics (FMM bag^−1^ and FMM PBM^−1^) showed greater discriminatory capacity than the volumetric metric (FVM bag^−1^), forming four distinct groups, while volume differentiated only two. Fruit mass per bag ranged from 205 to 252 kg bag^−1^, corresponding to a 22% range between individual extreme values. This range represents a direct impact on processing, as less efficient materials require greater fresh fruit volume to achieve the same final bean yield.

The proposed hypothesis was partially confirmed, since yield efficiency was strongly associated with fruit structural composition, particularly bean proportion, and was better captured by mass-based metrics. However, the absence of genetic variation for the volume-to-mass ratio indicates that efficiency differences arise predominantly from biomass partitioning between bean and husk, and not from variations in fruit structural density.

## Figures and Tables

**Table 1:** Summary of analysis of variance for FMM bag^−1^, FVM bag^−1^, FMM PBM^−1^, and FVM FMM^−1^ in *Coffea canephora* genotypes, with mean squares for genotypes, coefficients of variation (CV, %), and overall means of traits.

**Source of Variation**	**Mean Squares**						
	**df**	**FMM Bag^−1^**	**FVM Bag^−1^**	**FMM PBM^−1^**	**FVM FMM^−1^**	**% Bean**	**% Husk**
Blocks	2	10.5557	226.0969	0. 0029	0. 0043	1.9105	0. 9553
Genotypes	47	673.282 **	1975.9181 **	0.1870 **	0.0063 ns	930.0677 **	19.7887 **
Error	94	103.4343	732.4387	0. 0288	0.0155	172.1906	1.8318
Mean		223.84	356.25	3.73	1.59	55.33	44.67
CV (%)		4.54	7.60	4.55	7.82	2.45	3.03

** and ns indicate significant and non-significant effects, respectively, by the F-test at 5% probability. FMM Bag^−1^: mass of ripe fruit per 60 kg bag of processed coffee; FVM Bag^−1^: volume of ripe fruit per 60 kg bag of processed coffee; FMM PBM ^−1^: mass of ripe fruit per mass of processed beans; FVM FMM^−1^: ratio of fruit volume to fruit mass; CV: coefficient of variation.

**Table 2:** Estimates of genetic parameters for FMM bag^−1^, FVM bag^−1^, FMM PBM^−1^, FVM FMM^−1^, bean proportion, and husk proportion in *Coffea canephora* genotypes.

Trait	Genetic Parameters					
	σ^2^ph	σ^2^g	σ^2^e	h^2^ (%)	CVg (%)	CVg/CVe
FMM bag^−1^	224.4274	189.9493	34.4781	84.64	6.16	1.35
FVM bag^−1^	658.5394	414.3931	244.1462	62.93	5.71	0.75
FMM PBM^−1^	0.0623	0.0527	0.0096	84.58	6.15	1.35
FVM FMM^−1^	0.0021	0.000	0.0052	-	-	-
% Bean	6.5962	5.9856	0.6106	90.74	4.42	1.81
% Husk	6.5962	5.9856	0.6106	90.74	5.48	1.81

**Table 3:** *Coffea canephora* genotypes evaluated (40 hybrids and eight Conilon), with group identification and commonly identified and/or registered name.

Genotype	Group	Genotype	Group	Genotype	Group
Peneirão	Conilon	AS5	Hybrid	3	Hybrid
Clementino	Conilon	R22	Hybrid	Pé de Ouro	Hybrid
Pirata	Conilon	VR1	Hybrid	VR5	Hybrid
AD1	Conilon	JC221	Hybrid	RMD	Hybrid
K61	Conilon	VR11	Hybrid	G1	Hybrid
CM1	Conilon	G4	Hybrid	110	Hybrid
Z21	Conilon	R08	Hybrid	RG2	Hybrid
L80	Conilon	PDP	Hybrid	6	Hybrid
A1	Hybrid	AS2	Hybrid	VR2	Hybrid
180	Hybrid	41	Hybrid	101	Hybrid
10	Hybrid	G30	Hybrid	B11	Hybrid
LB33	Hybrid	LB15	Hybrid	AS12	Hybrid
VR3	Hybrid	25	Hybrid	B12	Hybrid
AS1	Hybrid	29	Hybrid	7	Hybrid
LB80	Hybrid	G20	Hybrid	G2	Hybrid
AS6	Hybrid	VR4	Hybrid	Neguim	Hybrid

## Data Availability

The original contributions presented in this study are included in the article. Further inquiries can be directed to the corresponding authors.

## Abbreviations

The following abbreviations are used in this manuscript:

ANOVA	Analysis of variance
CV	Coefficient of variation
CVe	Environmental coefficient of variation
CVg	Genotypic coefficient of variation
EMMs	Estimated marginal means
FMM	Fresh fruit mass
FVM	Fresh fruit volume
h^2^	Heritability on a family mean basis
IDE	Integrated development environment
PBM	Processed bean mass
PCA	Principal component analysis
UPGMA	Unweighted Pair Group Method with Arithmetic Mean
σ^2^e	Environmental variance
σ^2^g	Genotypic variance
σ^2^ph	Phenotypic variance
